# Combination of C_v-a_CO_2_/C_a-v_O_2_ and P_v-a_CO_2_ as markers of resuscitation or microcirculation in patients with septic shock: a pilot study

**DOI:** 10.1186/s40560-025-00801-2

**Published:** 2025-06-17

**Authors:** Luping Cheng, Wenxin Wang, Xia Hu, Chuanliang Pan

**Affiliations:** 1 North Sichuan Medical College, Nanchong, Sichuan China; 2 Department of Intensive Care Unit, The Third People’s Hospital of Chengdu, Affiliated Hospital of Southwest Jiaotong University, Chengdu, China

**Keywords:** Septic shock, Microcirculation, Oxygen consumption, Perfusion, Mortality

## Abstract

**Background:**

The ratio of central venous-to-arterial carbon dioxide content difference to arterial-to-venous oxygen content difference (C_v-a_CO_2_/C_a-v_O_2_) and central venous-to-arterial carbon dioxide tension difference (P_v-a_CO_2_) are indicators for monitoring anaerobic metabolism and tissue perfusion in shock. We hypothesized that significant differences in patient outcomes exist across different C_v-a_CO_2_/C_a-v_O_2_ and P_v-a_CO_2_ groups during the early stages of shock resuscitation and that these two indicators reflect microcirculatory perfusion in septic shock patients.

**Methods:**

This single-center, prospective, observational, cohort, exploratory, pilot study involved newly diagnosed patients with septic shock admitted to intensive care unit (ICU) between May 2023 and August 2024. We classified patients into four groups based on their C_v-a_CO_2_/C_a-v_O_2_ and P_v-a_CO_2_ levels at 6 h post-ICU admission (T6), monitored sublingual microcirculation, and followed them for 28 days. The grouping is as follows: Group A is C_v-a_CO_2_/C_a-v_O_2_ ≤ 1 and P_v-a_CO_2_ < 6 mmHg; Group B is C_v-a_CO_2_/C_a-v_O_2_ ≤ 1 and P_v-a_CO_2_ ≥ 6 mmHg; Group C is C_v-a_CO_2_/C_a-v_O_2_ > 1 and P_v-a_CO_2_ < 6 mmHg; and Group D is C_v-a_CO_2_/C_a-v_O_2_ > 1 and P_v-a_CO_2_ ≥ 6 mmHg.

**Results:**

105 patients were included in the study. The 28-day mortality differed significantly among the four groups of patients (A:8.3%, B:19%, C:30%, and D:46.7%, *p* < 0.05). The Kaplan–Meier curves for the four groups revealed significant differences in the 28-day survival probabilities. (*p* = 0.014). Multivariate Cox regression revealed that the independent risk factors for 28-day mortality were age [hazard ratio (HR) = 1.05, 95% confidence interval (95% CI) = 1.02–1.09, *p* = 0.001], C_v-a_CO_2_/C_a-v_O_2_ (HR = 1.67, 95% CI = 1.03–2.69, *p* = 0.036), and P_v-a_CO_2_ (HR = 1.13, 95% CI = 1.00–1.27, *p* = 0.043). There were significant differences among the four groups in terms of the proportion of perfused vessels for all (PPV), proportion of perfused vessels for *d* < 20 μm (sPPV), microvascular flow index (MFI), and heterogeneity index (HI) values (*p* < 0.001); correlations were observed for C_v-a_CO_2_/C_a-v_O_2_, P_v-a_CO_2_, and sPPV (*r* = −0.49, *p* < 0.001, R^2^ = 0.19; *r* = −0.22, *p* = 0.028, R^2^ = 0.08).

**Conclusions:**

The combined assessment of C_v-a_CO_2_/C_a-v_O_2_ and P_v-a_CO_2_ during the early stages of resuscitation demonstrates a significant association with mortality in septic shock patients. This combination could potentially serve as a resuscitation target and reflect microcirculatory perfusion in septic shock patients.

**Supplementary Information:**

The online version contains supplementary material available at 10.1186/s40560-025-00801-2.

## Background

Septic shock is a life-threatening condition with a high mortality rate of approximately 30% [1, 2]. In septic shock, a mismatch between systemic oxygen delivery (DO_2_) and tissue oxygen demand leads to tissue hypoperfusion, which contributes to oxygen debt, cellular dysfunction, and multi-organ failure, significantly increasing the risk of mortality. Tissue hypoxia and impaired perfusion are interdependent processes that drive the progression of critical illness. Early identification of inadequate tissue perfusion and timely resuscitation during the initial phase of septic shock are critical for improving outcomes [3], with the ratio of central venous-to-arterial carbon dioxide content difference to arterial-to-venous oxygen content difference (C_v-a_CO_2_/C_a-v_O_2_) and the central venous-to-arterial carbon dioxide tension difference (P_v-a_CO_2_) emerging as promising markers for this purpose.

Various biomarkers have been proposed for assessing hypoxia and tissue perfusion. Lactate is widely used, though its prognostic value remains controversial [4–6]. Venous O_2_ saturation (S_v_O_2_) reflects the balance of oxygen supply and demand but has shown limited benefit as a resuscitation target [1, 7, 8]. C_v-a_CO_2_/C_a-v_O_2_, derived from the Fick equation, reflects the ratio of carbon dioxide production (VCO2) to oxygen consumption (VO_2_) and serves as a surrogate marker for the respiratory quotient (RQ), independent of cardiac output (CO). C_v-a_CO_2_/C_a-v_O_2_ has been proposed as a marker for detecting tissue hypoxia in critically ill patients [9, 10], with sustained elevations being associated with unfavorable clinical outcomes. Conversely, the analogous ratio of central venous-to-arterial carbon dioxide tension difference to arterial-to-venous oxygen content difference (P_v-a_CO_2_/C_a-v_O_2_) has not demonstrated comparable prognostic significance [9]. However, C_v-a_CO_2_/C_a-v_O_2_ does not allow for differentiation between hypoxia resulting from inadequate tissue perfusion and that due to other causes. To more accurately assess the outcomes of patients with septic shock, finding biomarkers that more sensitively reflect tissue perfusion and hypoxia has become an urgent issue to be addressed. P_v-a_CO_2_ can reflect peripheral tissue perfusion [11] and cardiac output (CO) [12]. Vallet et al. [13] and Pischkes et al. [14] have demonstrated that elevated P_v-a_CO_2_ correlates with decreased blood flow. C_v-a_CO_2_/C_a-v_O_2_ and P_v-a_CO_2_ are more receptive indicators of early tissue perfusion and hypoxia during shock than lactate, because blood CO_2_ and O_2_ levels change more rapidly and sensitively [10]. C_v-a_CO_2_/C_a-v_O_2_ and P_v-a_CO_2_ are considered more sensitive indicators for monitoring cellular hypoxia and tissue perfusion, respectively, during the early stages of shock. Additionally, There is a marked dissociation between macrocirculatory parameters and those reflecting microcirculatory perfusion in the context of septic shock [15, 16]. Microcirculation plays a pivotal role in the pathogenesis of organ dysfunction in sepsis, and sidestream dark field (SDF) imaging enables real-time visualization of microcirculation, providing insights into perfusion at the capillary and small vessel level [17].

Previous studies have investigated either one of these two indicators individually or in combination with other parameters to assess prognosis and guide resuscitation in patients with septic shock. However, there is a paucity of research evaluating the prognostic value of combining both C_v-a_CO_2_/C_a-v_O_2_ and P_v-a_CO_2_ in the early stages of septic shock. And the potential relationship between these two indicators and microcirculatory alterations remains unclear.

Therefore, the primary objective of this study is to examine the association between the combined use of C_v-a_CO_2_/C_a-v_O_2_ and P_v-a_CO_2_ and clinical outcomes in patients with septic shock.The secondary objective is to explore the relationship between these two parameters and sublingual microcirculation parameters to comprehensively evaluate the association between macro- and micro-hemodynamics, ultimately guiding more effective and individualized resuscitation strategies for septic shock patients. We hypothesize that the prognosis of patients differs significantly among groups stratified by C_v-a_CO_2_/C_a-v_O_2_ and P_v-a_CO_2_ during the early phase of shock resuscitation, and that these two indicators reflect microcirculatory perfusion in septic shock patients.

## Methods

### Study design, patients, and ethics

This pilot study involved newly diagnosed septic shock patients admitted to the Third People’s Hospital of Chengdu’s intensive care unit (ICU) between May 2023 and August 2024. These patients underwent endotracheal intubation and had superior vena cava and arterial catheters placed as needed.

The diagnosis of septic shock followed the Sepsis-3 criteria [18], with the diagnostic process as follows: For patients with infection or suspected infection, if their sequential organ failure assessment (SOFA) score was ≥2 points or if the clinician suspected sepsis, further evaluation for evidence of organ dysfunction was conducted. If the patient’s SOFA score was ≥2 points or increased by ≥2 points, they were diagnosed with sepsis. Patients with sepsis who required vasopressors to maintain a mean arterial pressure (MAP) ≥ 65 mmHg despite adequate fluid resuscitation, and who had a lactate level >2 mmol/L, were diagnosed with septic shock.

We included septic shock patients aged 18–85 years old who met central venous pressure (CVP) ≥ 8 mmHg after appropriate resuscitation, MAP ≥ 65 mmHg under norepinephrine infusion (≥0.02 µg/kg/min), and lactate >2 mmol/L at 6 h post-ICU admission (T6). This not only ensures compliance with the diagnostic criteria for septic shock but also excludes patients who may be in states of over-resuscitation or under-resuscitation, thereby guaranteeing the safety and accuracy of the study results. Pregnant women, those with severe chronic obstructive pulmonary disease, limited treatment effort, a previous episode of sepsis or septic shock within the last 3 months, malignant tumors, or severe cirrhosis (Child–Pugh type C) were excluded.

The hospital’s Medical Ethics Review Committee approved the study, which followed the medical ethics rules and the Declaration of Helsinki. (Approval number [2023] S–14). Informed consent was obtained from patients or their relatives. This study followed the “Strengthening the Reporting of Observational Studies in Epidemiology (STROBE)” statement guidelines [19] for observational cohort studies.

### General management

Within 6 h after ICU admission, all patients received a comprehensive treatment protocol emphasizing early shock resuscitation and hemodynamic optimization. The resuscitation targets included a cardiac index (CI) > 3 L/min/m^2^, measured by bedside ultrasound or thermodilution, and a MAP ≥ 65 mmHg, maintained using norepinephrine as the preferred vasopressor. Additional targets included CVP ≥ 8 mmHg, a central venous O_2_ saturation (S_cv_O_2_) ≥ 70%, and urine output >0.5 ml/kg/h. Respiratory support was provided primarily in volume-controlled/auxiliary mode, with target tidal volume of 6–8 ml/kg [ideal body weight (IBW)] or lower, plateau pressure (Pplat) < 30 cmH_2_O, and driving pressure <15 cmH_2_O. Positive end-expiratory pressure (PEEP) titrated based on fraction of inspiration O_2_ (FiO_2_) [the acute respiratory distress syndrome (ARDS) Network lower PEEP table [20]].Target partial pressure of O_2_ in arterial blood (P_a_O_2_) and partial pressure of CO_2_ in arterial blood (P_a_CO_2_) be 70–100 and 35–45 mmHg, respectively. For sedation and analgesia, dexmedetomidine or midazolam was administered via continuous intravenous infusion for sedation, and fentanyl or butorphanol for analgesia, aiming for the Critical-Care Pain Observation Tool (CPOT) scores <3 points and the early sedation target of −3 points. Empirical antibiotic therapy was initiated following timely collection of microbiological cultures. Glycemic control was achieved with insulin infusion to maintain blood glucose at 7.8–10.0 mmol/L (140–180 mg/dL;1 mmol/L = 18 mg/dL). Lactate levels were dynamically monitored, and acid–base and electrolyte imbalances were corrected. Anemia was managed with transfusion targeting hemoglobin (Hb) > 7.0 g/dL or hematocrit (Hct) > 0.22], etc.

### Study protocol and follow up

Time 0 (T0) was defined as the moment of the patients’ admission to the ICU. At T6, we collected blood samples for arterial and central venous (the superior vena cava) gas analyses (i-STAT 1 Portable Clinical Analyzer, Abbott, Chicago, USA.). Arterial lactate, MAP, CVP, heart rate (HR), volume of fluid resuscitation, FiO_2_ of the ventilator, PEEP, body temperature (BT), Hb concentration, and pressor agent dosage [vasoactive-inotropic score (VIS) [21]] were monitored. we also monitored the sublingual microcirculation at T6.

We recorded the SOFA scores, Acute Physiology and Chronic Health Evaluation II (APACHE II) scores, and lactate at T6, 24 h (T24), 48 h (T48), and 72 h (T72) post-ICU admission, along with endotracheal intubation duration, ICU length of stay, and in-hospital mortality. We tracked and recorded the survival status of each patient for 28 days from the time of enrollment, regardless of whether they were still receiving treatment at our hospital or another medical facility. We also calculated the ventilator-free days and ICU-free days. Ventilator-free days refer to the number of days a patient has not been intubated during the period from admission to the ICU until the end of the 28-day follow-up; ICU-free days refer to the number of days a patient has not received ICU treatment within the same timeframe.

### Sublingual microcirculatory measurements and calculations

We used SDF imaging equipment (Microsense V100; Yiruan Intelligent Technology Co., Guangzhou, China) to monitor the sublingual microcirculation of patients at T6. After lightly removing saliva using gauze or swabs, the SDF probe was delicately positioned on one side of the tongue, 2.5–4 cm from the tip. The operator recorded five video clips from adjacent mucosal regions, with each segment lasting at least 20 s. All the video recordings were obtained by two specially trained and experienced non-attending physicians. Eligible video clips were anonymized by replacing patient identifiers with random numbers and were saved offline. We used the “Microcirculation Image Quality Score” described by Massey et al. [22] to systematically evaluate the quality of the collected images. Video sequences with poor quality (scores ≥ 10) were discarded at bedside. These videos were then analyzed in a blinded manner by another specialized researcher. The videos were analyzed using the software provided by the instrument.

Continuous blood flow in the microvessels is regarded as normal, whereas sluggish, intermittent, or no blood flow is considered abnormal. Microvessels are categorized as small or large, with a 20 μm diameter cutoff. Following the consensus [23], we calculated the proportion of perfused vessels for *d* < 20 μm (sPPV), proportion of perfused vessels for all (PPV), microcirculatory flow index (MFI), heterogeneity index (HI), total vessel density (TVD), vessel (*d* < 20 μm) density (sVD), perfused vessel density (PVD), and perfused vessel (*d* < 20 μm) density (sPVD). The proportion of perfused vessels (by length) is defined as the total length of perfused vessels (L_p_) divided by the sum of the lengths of all vessels (L_v_), expressed as PPV = L_p_/L_v_, with a unit of %. The perfused vessel density is defined as the L_p_ divided by the total analyzed field of view area (A_FOV_), that is PVD = L_p_/A_FOV_, in mm/mm^2^.

### Formulas and calculations

In accordance with the Fick equation:

1 $$ {\text{VO}}_{2} = {\text{CO}} \times ({\text{C}}_{{\text{a}}} {\text{O}}_{2} - {\text{C}}_{{\text{v}}} {\text{O}}_{2} ) $$

2 $$ {\text{VCO}}_{2} = {\text{CO}} \times ({\text{C}}_{{\text{v}}} {\text{CO}}_{2} - {\text{C}}_{{\text{a}}} {\text{CO}}_{2} ) $$

VO_2_, oxygen consumption; CO, cardiac output; C_a_O_2_, arterial oxygen content; C_v_O_2_, venous oxygen content; VCO_2_, CO_2_ production; C_v_CO_2_, venous blood CO_2_ content; C_a_CO_2_, arterial blood CO_2_ content.

From Eqs. (1) and (2), it follows that:

3 $$ {\text{VCO}}_{2} /{\text{VO}}_{2} = ({\text{C}}_{{\text{v}}} {\text{CO}}_{2} - {\text{C}}_{{\text{a}}} {\text{CO}}_{2} )/({\text{C}}_{{\text{a}}} {\text{O}}_{2} - {\text{C}}_{{\text{v}}} {\text{O}}_{2} ) $$

In Eq. (3), VCO_2_/VO_2_ represents the respiratory quotient (RQ). Therefore, (C_v_CO_2_–C_a_CO_2_)/(C_a_O_2_–C_v_O_2_) can be used as an alternative indicator of RQ.

$$ {\text{C}}_{{\text{a}}} {\text{O}}_{2} = ({\text{Hb}} \times {\text{S}}_{{\text{a}}} {\text{O}}_{2} \times 1.34) + ({\text{P}}_{{\text{a}}} {\text{O}}_{2} \times 0.0031) $$

$$ {\text{C}}_{{\text{v}}} {\text{O}}_{2} = ({\text{Hb}} \times {\text{S}}_{{\text{v}}} {\text{O}}_{2} \times 1.34) + ({\text{P}}_{v} {\text{O}}_{2} \times 0.0031) $$

Hb, hemoglobin; SaO_2_, arterial oxygen saturation; P_a_O_2_, arterial oxygen partial pressure; S_v_O_2_, venous oxygen saturation; P_v_O_2_, venous oxygen partial pressure.

The calculation of blood CO_2_ content follows the Douglas formula [24]:

$$ \begin{aligned} & {\text{Blood}}\;{\text{CO}}_{2} \;{\text{content}} = {\text{plasma}}\;{\text{CO}}_{2} \;{\text{content}} \times \left( {1 - 0.0289 \times {\text{Hb}}} \right) \div \left( {3.352 - 0.456 \times {\text{SO}}_{2} } \right) \times \left( {8.142 - {\text{pH}}} \right) \\ & {\text{Plasma}}\;{\text{CO}}_{2} \;{\text{content}} = 2.226 \times {\text{Plasma}}\;{\text{CO}}_{2} \;{\text{solubility}} \times {\text{plasma}}\;{\text{PCO}}_{2} \times \left( {1 + 10^{{{\text{pH}} - {\text{pK}}\prime }} } \right) \\ & {\text{Plasma}}\;{\text{CO}}_{2} \;{\text{solubility}} = 0.0307 + 0.00057 \times \left( {37 - {\text{T}}} \right) + 0.00002 \times \left( {37 - {\text{T}}} \right)^{2} \\ & {\text{pK}}\prime = 6.086 + 0.042 \times \left( {7.4 - {\text{pH}}} \right) + \left( {38 - {\text{T}}} \right) \times \left[ {0.00472 + 0.00139 \times \left( {7.4 - {\text{pH}}} \right)} \right] \\ \end{aligned} $$

pH, potential of hydrogen; BT, blood temperature.

VIS [21] will be calculated as follows: VIS [21] = dopamine dose (µg/kg/min) + dobutamine dose (µg/kg/min) + 100 × epinephrine dose (µg/kg/min) + 100 × norepinephrine dose (µg/kg/min) + 15 × milrinone dose (µg/kg/min) + 10,000 × vasopressin dose (U/kg/min).

### Definitions of the four groups

Given that C_v-a_CO_2_/C_a-v_O_2_ and P_v-a_CO_2_ serve as reliable indicators of anaerobic metabolism and tissue perfusion, respectively. The C_v-a_CO_2_/C_a-v_O_2_ > 1 was reported to be both sensitive and specific for detecting anaerobic metabolism in critically ill patients [25]. Previous study [26] reported that under physiological conditions, P_v-a_CO_2_ remains below 6 mmHg. So, we classified patients into four distinct groups based on their C_v-a_CO_2_/C_a-v_O_2_ and P_v-a_CO_2_ levels at T6: Group A was no anaerobic metabolism and no inadequate tissue perfusion, C_v-a_CO_2_/C_a-v_O_2_ ≤ 1 and P_v-a_CO_2_ < 6 mmHg; Group B was no anaerobic metabolism but inadequate tissue perfusion, C_v-a_CO_2_/C_a-v_O_2_ ≤ 1 and P_v-a_CO_2_ ≥ 6 mmHg; Group C was anaerobic metabolism but no inadequate tissue perfusion, C_v-a_CO_2_/C_a-v_O_2_ > 1 and P_v-a_CO_2_ < 6 mmHg; and Group D was anaerobic metabolism and inadequate tissue perfusion, C_v-a_CO_2_/C_a-v_O_2_ > 1 and P_v-a_CO_2_ ≥ 6 mmHg.

## Outcome

The primary outcome of this study was 28-day mortality, with secondary outcomes including the duration of endotracheal intubation, ICU length of stay, ventilator-free days, ICU-free days, in-hospital mortality, and microcirculation.

### Sample size estimation

Our hypothesis for the primary outcome is that there is a significant difference in 28-day mortality among patients in different levels of the Cv-aCO_2_/Ca-vO_2_ and Pv-aCO_2_ subgroups. Based on the study design and relevant literature [27], the sample size calculation was performed under the following parameters: a significance level of *α* = 0.05, a statistical power of 80% (*β* = 0.20), and assumed mortality rates of *p*_0_ = 0.023 and *p*_1_ = 0.30 between groups. Using SPSS software, the results indicated that a minimum of 26 participants per group is required, resulting in a total sample size of at least 105 participants. 105 patients would provide a good correlation between the subgroups and 28-day mortality.

## Statistics

Data are expressed as number (%) for categorical and as mean (standard deviation, ±SD) or median (interquartile range, IQR) for continuous variables. Comparisons among groups of categorical variables were performed using the chi-square (χ^2^) or Fisher’s exact test. Normally distributed tests were conducted using Kolmogorov-Smirnoff (n > 50) or Shapiro–Wilk (n ≤ 50) analyses. Non-normally distributed continuous variables were compared among the groups using the Kruskal–Wallis test. Intergroup comparisons were performed using an analysis of variance (ANOVA) for normally distributed continuous variables. The Bonferroni test was employed for the post-hoc pairwise comparison, and repeated-measures data, such as lactate levels and SOFA and APACHE II scores, were compared among the groups using repeated-measures ANOVA. P_v-a_CO_2_, C_v-a_CO_2_/C_a-v_O_2,_ and sPPV were also evaluated for correlation at T6 using the Spearman’s rho test and R^2^. The Kaplan–Meier method was employed to estimate the 28-day survival curves. We used the log-rank (Mantel-Cox) test to determine group differences. We assessed the predictive variables for 28-day mortality and generated hazard ratios (HR) using univariate and multivariate Cox regressions. Differences with a *P* value of less than 0.05 were considered statistically significant. Data analysis was performed using SPSS version 26 software (IBM, New York, USA).

## Results

**Fig. 1:**
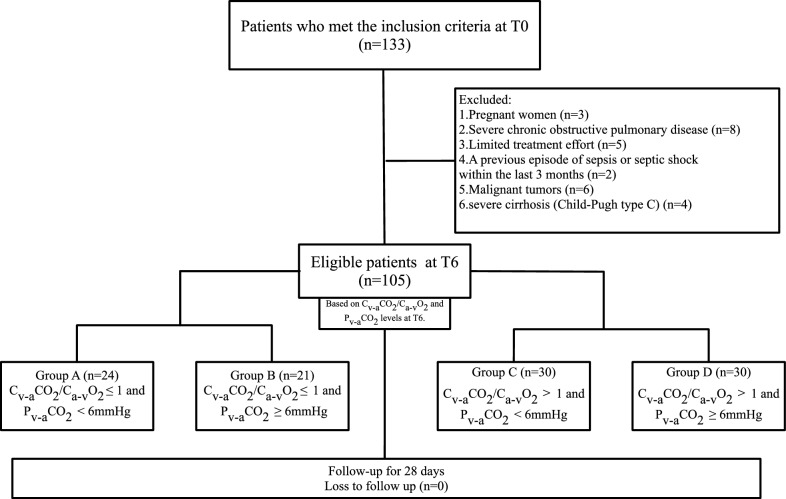
Patient flowchart. 133 patients met the inclusion criteria, then excluded patients who did not meet the criteria, and finally 105 patients were included in the study. According to different C_v-a_CO_2_/C_a-v_O_2_ and P_v-a_CO_2_ levels at T6, they were divided into Group A (C_v-a_CO_2_/C_a-v_O_2_ ≤ 1 and P_v-a_CO_2_ < 6 mmHg), Group B (C_v-a_CO_2_/C_a-v_O_2_ ≤ 1 and P_v-a_CO_2_ ≥ 6 mmHg), Group C (C_v-a_CO_2_/C_a-v_O_2_ > 1 and P_v-a_CO_2_ < 6 mmHg) and Group D (C_v-a_CO_2_/C_a-v_O_2_ > 1 and P_v-a_CO_2_ ≥ 6 mmHg), with 24 participants in Group A, 21 participants in Group B, 30 participants in Group C and 30 participants in Group D

The patient selection process is illustrated in Fig. 1. The 15-month study period included 105 patients. 28-day and in-hospital mortality rates were 27.6 and 21.9%, respectively. Endotracheal intubation duration lasted 5.0 (3.0, 8.0) days, ICU length of stay was 9.0 (6.0, 13.0) days, ventilator-free days was 21.0 (14.0, 24.0) days, and ICU-free days lasted 17.0 (9.5, 21.0) days (Table 2).

Patients’ general characteristics are shown in Table 1. Lung infections (42.9%) and use of internal medicine (76.2%) dominated the study population. At T6, 18 patients (17.1%) underwent continuous renal replacement treatment (CRRT) following resuscitation. The median pre-T6 fluid volume was 3,374 (2,771, 3,855) ml. MAP: 70.0 (69.0, 73.5) mmHg; VIS: 25.9 ± 7.5; S_v_O_2_: 0.65 ± 0.13; lactate: 5.2 ± 2.0 mmol/L; APACHE II: 24.1 ± 4.5; SOFA: 11.4 ± 2.5 at T6. At T6, patients’ median C_v-a_CO_2_/C_a-v_O_2_ was 1.28 (0.62, 1.92), and the median P_v-a_CO_2_ was 5.8 (2.8, 9.2) mmHg. From this, 24 patients were assigned to Group A, 21 to B, 30 to C, and 30 to D. The patients’ general characteristics were not statistically different (*p* > 0.05).

**Table 1:** General characteristics

Variables	All patients (n = 105)	Group A (n = 24)	Group B (n = 21)	Group C (n = 30)	Group D (n = 30)	*P*
Age (years)	59.9 ± 13.6	61.5 ± 13.2	56.0 ± 14.5	61.4 ± 12.7	59.7 ± 14.1	0.499
Male (%)	46 (43.8)	12 (50.0)	9 (42.8)	13 (43.3)	12 (40.0)	0.905
BMI (kg/m^2^)	28.1 ± 4.7	28.3 ± 4.8	28.5 ± 5.1	27.7 ± 3.4	28.2 ± 5.6	0.930
Source of infection n (%)						
Pneumonia	45 (42.9)	14 (58.3)	8 (38.1)	13 (43.3)	10 (33.3)	0.236
Abdominal	25 (23.8)	2 (8.3)	5 (23.8)	8 (26.7)	10 (33.3)	
Urinary	13 (12.4)	3 (12.5)	2 (9.5)	5 (16.7)	3 (10.0)	
Soft tissue	6 (5.7)	3 (12.5)	2 (9.5)	0 (0)	1 (3.3)	
No specific site	9 (8.6)	2 (8.3)	2 (9.5)	1 (3.3)	4 (13.3)	
Other	7 (6.7)	0 (0)	2 (9.5)	3 (10.0)	2 (6.7)	
Medical /surgical n (%)	80 (76.2) / 25 (23.8)	19 (79.2) / 5 (20.8)	16 (76.2) / 5 (23.8)	22 (73.3) / 8 (26.7)	23 (76.7) / 7 (23.3)	0.968
Diabetes, n (%)	55 (52.4)	15 (62.5)	11 (52.4)	15 (50)	14 (46.7)	0.695
Hypertension, n (%)	65 (61.9)	16 (66.7)	13 (61.9)	18 (60.0)	18 (60.0)	0.956
CKD, n (%)	15 (14.3)	4 (16.7)	3 (14.3)	5 (16.7)	3 (10.0)	0.865
Cardiopathy, n (%)	16 (15.2)	3 (12.5)	3 (14.3)	4 (13.3)	6 (20.0)	0.864
T6, CRRT, n (%)	18 (17.1)	4 (16.7)	4 (19.0)	6 (20.0)	4 (13.3)	0.908
Fluids before T6 (ml)	3374 (2771, 3855)	3470 (2927, 3976)	3496 (3200, 3909)	3134 (2596, 3656)	3198 (2338, 3877)	0.184
T6, BT (℃)	37.5 (36.7, 38.6)	37.5 (36.9, 38.8)	37.4 (36.8, 38.4)	37.7 (36.5, 38.8)	37.7 (36.6, 38.3)	0.931
T6, MAP (mmHg)	70.0 (69.0, 73.5)	71.0 (69.0, 73.0)	70.0 (68.5, 73.0)	70.0 (69.0, 74.0)	70.5 (67.8, 73.2)	0.895
T6, CVP (mmHg)	11.0 (10.0, 13.0)	12.0 (10.0, 13.0)	11.0 (9.5, 12.0)	12.0 (10.0, 13.0)	11.0 (10.0, 12.0)	0.658
T6, HR (beats/min)	98.0 ± 13.2	103.3 ± 10.1	95.6 ± 11.8	95.7 ± 13.2	97.8 ± 15.4	0.138
T6, PEEP (cmH_2_O)	8.0 (6.5, 10.0)	7.0 (6.0, 9.8)	8.0 (6.5, 10.0)	9.0 (7.0, 11.2)	8.0 (7.0, 10.2)	0.053
T6, FiO_2_	0.51 (0.32, 0.72)	0.50, (0.30, 0.76)	0.48 (0.33, 0.78)	0.57 (0.39, 0.77)	0.48 (0.31, 0.66)	0.765
T6, Hb (g/L)	99.5 ± 15.6	98.9 ± 14.1	100.5 ± 13.7	103.6 ± 16.6	95.1 ± 16.4	0.206
T6, VIS	25.9 ± 7.5	25.7 ± 8.1	27.0 ± 6.8	26.8 ± 8.0	24.3 ± 7.1	0.512
T6, S_v_O_2_	0.65 ± 0.13	0.64 ± 0.14	0.66 ± 0.12	0.69 ± 0.13	0.62 ± 0.14	0.201
T6, lactate (mmol/L)	5.2 ± 2.0	5.1 ± 1.7	4.8 ± 1.8	5.2 ± 2.3	5.5 ± 2.2	0.695
T6, APACHE II	24.1 ± 4.5	23.5 ± 4.6	26.3 ± 3.4	23.1 ± 4.7	24.1 ± 4.7	0.084
T6, SOFA	11.4 ± 2.5	10.6 ± 2.4	11.0 ± 2.0	11.9 ± 2.2	11.7 ± 3.1	0.184

Patients were separated into four groups according to C_v-a_CO_2_/C_a-v_O_2_ and P_v-a_CO_2_ measured at T6: Group A, C_v-a_CO_2_/C_a-v_O_2_ ≤ 1 and P_v-a_CO_2_ < 6 mmHg; Group B, C_v-a_CO_2_/C_a-v_O_2_ ≤ 1 and P_v-a_CO_2_ ≥ 6 mmHg; Group C, C_v-a_CO_2_/C_a-v_O_2_ > 1 and P_v-a_CO_2_ < 6 mmHg; Group D, C_v-a_CO_2_/C_a-v_O_2_ > 1 and P_v-a_CO_2_ ≥ 6 mmHg

*T6* at 6 h post-ICU admission, *BMI* body mass index, *CKD* Chronic Kidney Disease, *CRRT* continuous renal replacement treatment, *MAP* mean arterial pressure, *CVP* central venous pressure, *HR* heart rate, *PEEP* positive end-expiratory pressure, *FiO*_*2*_ fraction of inspiration O_2_, *Hb* hemoglobin, *VIS* vasoactive-inotropic score, *S*_*v*_*O*_*2*_ central venous oxygen saturation, *APACHE II* acute physiology and chronic health evaluation II, *SOFA* sequential organ failure assessment

The lactate levels, SOFA scores, and APACHE II scores declined gradually from T6 to T72 (Table S1, Fig. S1), and the trends showed statistically significant differences among the groups (*p* < 0.01). Group A’s lactate levels and SOFA scores dropped faster compared to C and D (*p* < 0.05), and its APACHE II scores decreased only faster than in D (*p* < 0.05).

The clinical outcomes of the patients are shown in Table 2. Endotracheal intubation and ICU length of stay were not significantly different between groups. The four groups had significantly different in-hospital and 28-day mortality rates (*p* < 0.05). Group A had the lowest in-hospital and 28-day mortality, both at 8.3%; followed by Group B, with in-hospital mortality at 9.5% and 28-day mortality at 19.0%; and Group D had the highest rates, at 40 and 46.7%, respectively. There was a significant intergroup difference in ventilator-free days between Group A and Group D.

**Table 2:** Clinical outcomes

Variables	All patients (n = 105)	Group A (n = 24)	Group B (n = 21)	Group C (n = 30)	Group D (n = 30)	*P*
28-day mortality, n (%)	29 (27.6)	2 (8.3)	4 (19.0)	9 (30.0)	14 (46.7)	0.013
Duration of endotracheal intubation (days)	5.0 (3.0, 8.0)	4.5 (3.0, 8.0)	6.0 (3.0, 7.0)	5.0 (4.0, 8.2)	6.0 (3.0, 9.0)	0.552
Duration of ICU (days)	9.0 (6.0, 13.0)	7.0 (4.2, 13,0)	8.0 (5.0, 16.0)	9.0 (6.8, 12.0)	9.0 (6.0, 13.0)	0.766
Ventilator-free days (days)	21.0 (14.0, 24.0)	23.0 (19.2, 25.0)	22.0 (19.0, 24.0)	20.5 (11.2, 24.0)	19.0 (6.5, 22.5)	0.027*
ICU-free days (days)	17.0 (9.5, 21.0)	20.5 (14.2, 22.8)	15.0 (10.5, 21.5)	18.0 (5.5, 20.0)	15.0 (0, 19.5)	0.057
In-hospital mortality, n (%)	23 (21.9)	2 (8.3)	2 (9.5)	7 (23.3)	12 (40)	0.015

^*^ The post-hoc pairwise comparison using the Bonferroni method revealed a significant difference in Ventilator-free days between Group A and D. Group A, C_v-a_CO_2_/C_a-v_O_2_ ≤ 1 and P_v-a_CO_2_ < 6 mmHg; Group B, C_v-a_CO_2_/C_a-v_O_2_ ≤ 1 and P_v-a_CO_2_ ≥ 6 mmHg; Group C, C_v-a_CO_2_/C_a-v_O_2_ > 1 and P_v-a_CO_2_ < 6 mmHg; Group D, C_v-a_CO_2_/C_a-v_O_2_ > 1 and P_v-a_CO_2_ ≥ 6 mmHg

**Fig. 2:**
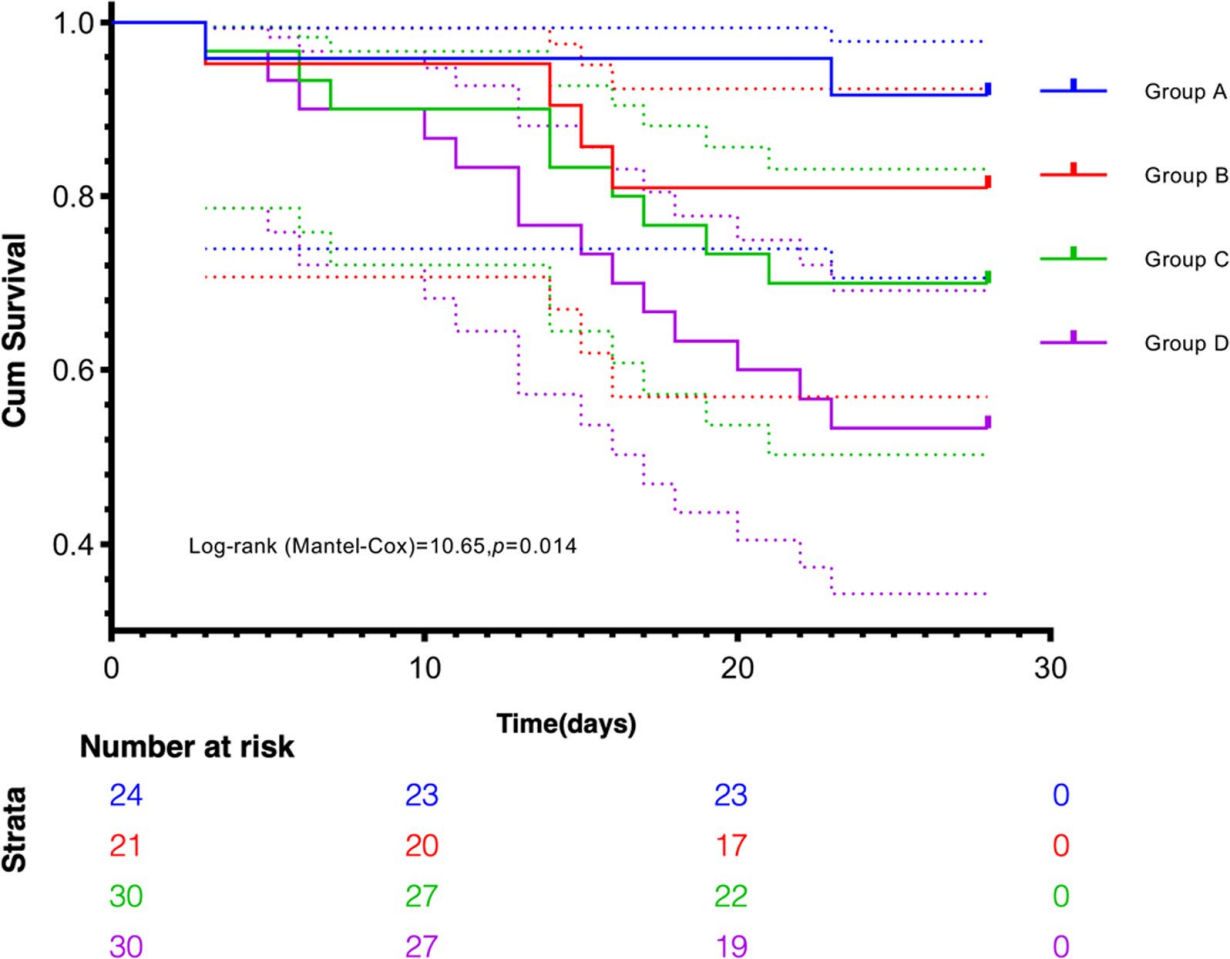
Survival probabilities up to day 28 according to P_v-a_CO_2_ and C_v-a_CO_2_/C_a-v_O_2_ at T6. Log-rank = 0.014. The dashed lines of the same hue on both sides of the survival curve represent their corresponding 95% confidence intervals

The four groups’ 28-day survival curves showed significant differences (*p* < 0.05), with Group A having the highest survival rate and Group D the lowest (Fig. 2). Univariate Cox regression of each baseline variable and microcirculatory indicator revealed that age, C_v-a_CO_2_/C_a-v_O_2_, P_v-a_CO_2_, and sPPV were risk factors for predicting 28-day mortality. Subsequently, the simultaneous inclusion of the four variables in the multivariate Cox regression revealed that age, C_v-a_CO_2_/C_a-v_O_2_, and P_v-a_CO_2_ were independent risk factors (Table S2, Fig. S2).

**Fig. 3:**
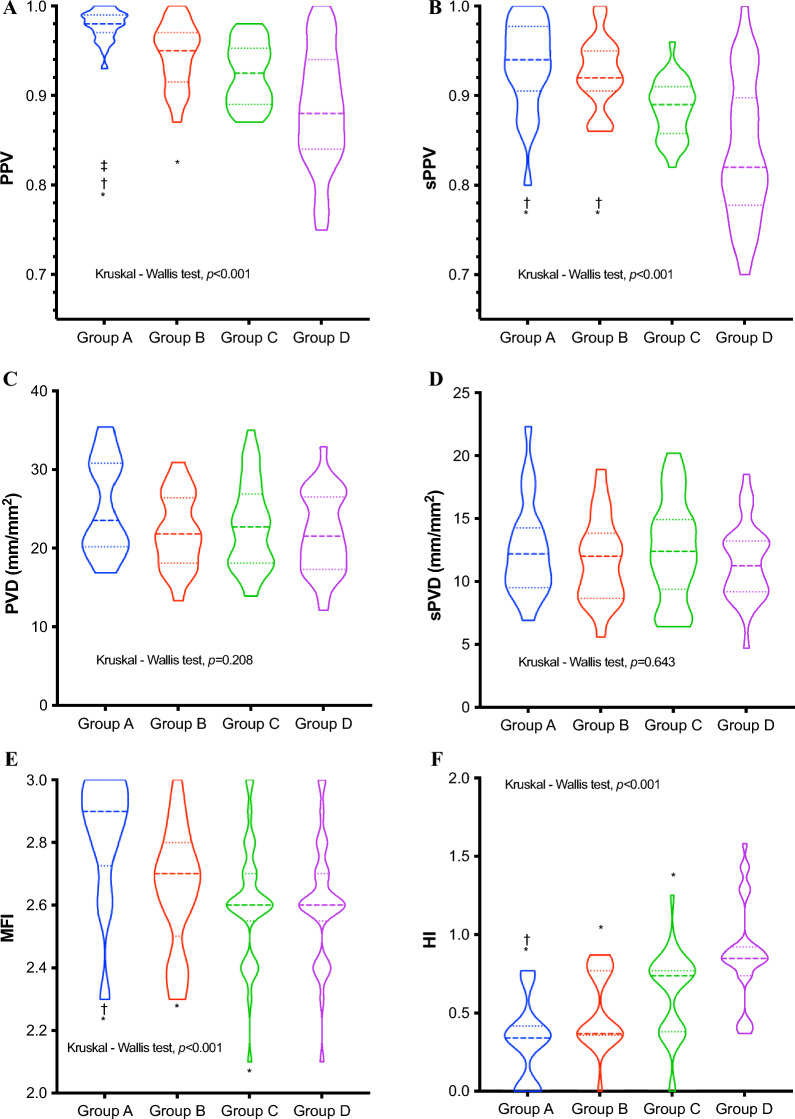
Violin plots depicting the differences in sublingual microcirculation indicators at T6. ‡*P* < 0.05 vs. Group B; †*P* < 0.05 vs. Group C; *P < 0.05 vs. Group D. The dashed horizontal lines indicate the quartiles (25th, 50th [median], and 75th percentiles). The Kruskal–Wallis test was used to compare the groups, assessing whether there is a statistically significant difference between their distributions. Patients were separated into four groups according to C_v-a_CO_2_/C_a-v_O_2_ and P_v-a_CO_2_ measured at T6: Group A, C_v-a_CO_2_/C_a-v_O_2_ ≤ 1 and P_v-a_CO_2_ < 6 mmHg; Group B, C_v-a_CO_2_/C_a-v_O_2_ ≤ 1 and P_v-a_CO_2_ ≥ 6 mmHg; Group C, C_v-a_CO_2_/C_a-v_O_2_ > 1 and P_v-a_CO_2_ < 6 mmHg; Group D, C_v-a_CO_2_/C_a-v_O_2_ > 1 and P_v-a_CO_2_ ≥ 6 mmHg. T6, at 6 h post-ICU admission; PPV, proportion of perfused vessels for all; sPPV, proportion of perfused vessels for *d* < 20 μm; PVD, perfused vessel density; sPVD, perfused vessel (d < 20 μm) density; MFI, microcirculatory flow index; HI, heterogeneity index

Sublingual microcirculation indicators at T6 are shown in Table S3 and Fig. 3. There were significant differences in PPV, sPPV, MFI, and HI values across the groups (*p* < 0.001). Group A had a far higher median PPV than the other three groups, with significant differences among the groups in post hoc comparisons (*p* < 0.05). Group A had the highest median sPPV value, followed by Group B, and both groups had higher than Groups C and D in the post hoc comparisons (*p* < 0.05). Group A had the highest median MFI, followed by Group B, and the lowest in Group D. Group A had the lowest median HI, followed by Group B, and the largest in Group D. Hence, Group A had much better microcirculatory than Group D, followed by Group B. Only the MFI and HI indicators were better in Group C than Group D.

**Fig. 4:**
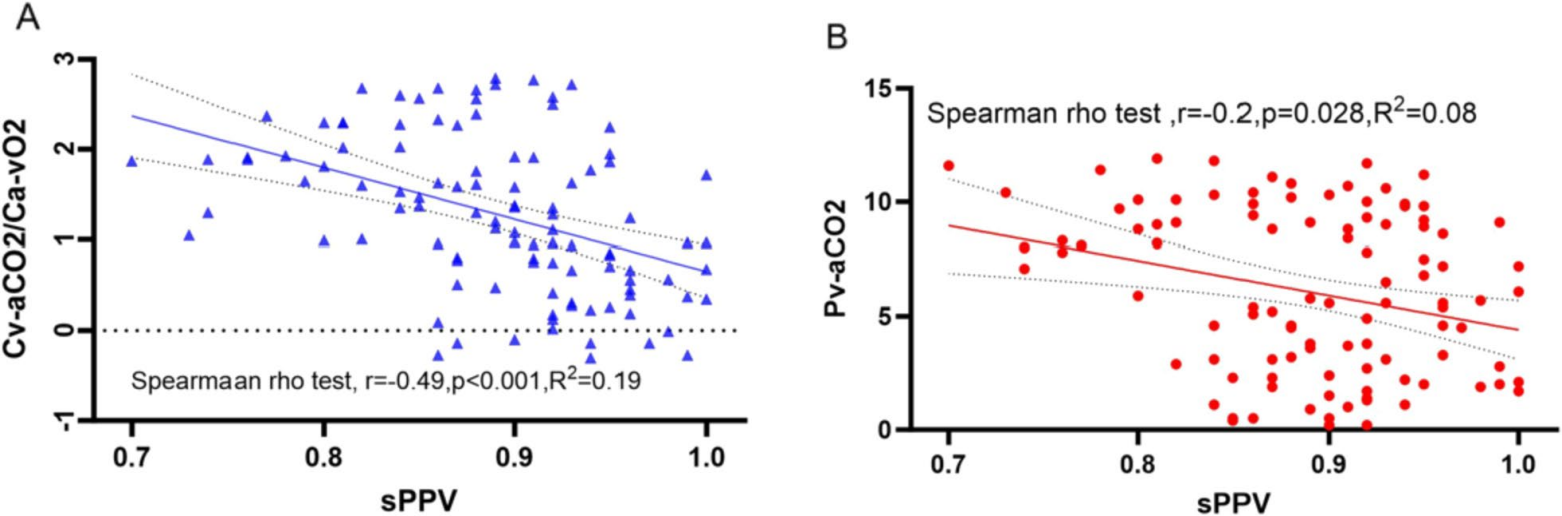
Scatter plots showing the relationships between the P_v-a_CO_2_, C_v-a_CO_2_/C_a-v_O_2_, and sPPV. The figure above shows two best-fit linear regression lines (solid) that lie within the 95% confidence band (dashed) respectively. sPPV, the proportion of perfused vessels for *d* < 20 μm

The association of sPPV with P_v-a_CO_2_ and C_v-a_CO_2_/C_a-v_O_2_ is shown in Fig. 4. Spearman’s rho test showed a correlation between the two and sPPV; subsequently, linear regression analysis was conducted (*p* < 0.05), which were relatively weak according to the correlation coefficient (*r*) and R^2^ values; however, C_v-a_CO_2_/C_a-v_O_2_ outperformed P_v-a_CO_2_.

## Discussion

This study discovered that 6 h after admission, elevated C_v-a_CO_2_/C_a-v_O_2_ and P_v-a_CO_2_ were associated with the poorest mortality. Conversely, patients who maintained both C_v-a_CO_2_/C_a-v_O_2_ and P_v-a_CO_2_ within normal ranges throughout the 6-h period after ICU admission were correlated with the best outcomes. We found that patients with C_v-a_CO_2_/C_a-v_O_2_ < 1 and P_v-a_CO_2_ ≥ 6 mmHg had better clinical outcomes than those with C_v-a_CO_2_/C_a-v_O_2_ > 1 and P_v-a_CO_2_ < 6 mmHg. C_v-a_CO_2_/C_a-v_O_2_ and P_v-a_CO_2_ were independent risk factors for predicting 28-day mortality in the multivariate Cox regression, which supports the findings of other studies [9, 26].

The preconditions for this study assumed that C_v-a_CO_2_/C_a-v_O_2_ and P_v-a_CO_2_ were reliable indicators of anaerobic metabolism and tissue perfusion, as well as two essential indicators of septic shock. VCO_2_ should not exceed VO_2_ under aerobic conditions, and C_v-a_CO_2_/C_a-v_O_2_, an alternative RQ indicator, should not exceed one. Thus, C_v-a_CO_2_/C_a-v_O_2_ > 1 can recognize the excess CO_2_ produced due to anaerobic metabolism [10]. These mechanisms may explain this study’s unfavorable clinical outcomes, including greater mortality in Group C and D. P_v-a_CO_2_/C_a-v_O_2_ is often used as a surrogate for C_v-a_CO_2_/C_a-v_O_2_ due to its clinical convenience [26, 28], but it is influenced by multiple factors [29, 30]. During early septic shock resuscitation, when the patient is in a non-steady state, the agreement between the two may be unreliable [9]. Therefore, this study used C_v-a_CO_2_/C_a-v_O_2_ to improve accuracy. Target values were quickly obtained using a pre-defined program requiring only Hb, pH, SO_2_, PCO_2_, and BT.

Theoretically, however, C_v-a_CO_2_/C_a-v_O_2_ is independent of systemic blood flow. Its combination with other tissue perfusion markers may offer enhanced insight during the early phase of septic shock. Recently, many studies [31, 32] have suggested that P_v-a_CO_2_ can be considered a potential biochemical indicator for evaluating inadequate tissue perfusion, indicating a possible correlation with tissue perfusion, and a normal P_v-a_CO_2_ value alone does not rule out the presence of tissue hypoxia [33]. Ospina-Tascón et al. [32] reported that P_v-a_CO_2_ is strongly correlated with microcirculatory indicators but not with CO in the early stage of septic shock. Therefore, some authors have suggested that P_v-a_CO_2_ reflects tissue perfusion. This study demonstrates that patients with simultaneous elevations in both C_v-a_CO_2_/C_a-v_O_2_ and P_v-a_CO_2_ exhibit the poorest prognosis, whereas those with normal values for both parameters have the most favorable outcomes. C_v-a_CO_2_/C_a-v_O_2_ > 1 combined with elevated P_v-a_CO_2_ may reflect ongoing hypoperfusion and persistent hypoxia, indicating the need to optimize both macro- and microcirculatory function. Conversely, C_v-a_CO_2_/C_a-v_O_2_ > 1 with normal P_v-a_CO_2_ may suggest hypoxia related to mitochondrial dysfunction, in which case further resuscitation may be unwarranted. This hypothesis warrants validation in future multicenter, large-scale prospective studies.

In the hemodynamic management of septic shock, the primary goals are to improve tissue perfusion and correct tissue hypoxia. The order of grouping (A → B → C → D) is as progressively worse as lactate, SOFA scores, APACHE II scores, microcirculation alterations, in-hospital mortality, and 28-day mortality, despite no significant differences across specific groups. The results of the study demonstrated that the combination of C_v-a_CO_2_/C_a-v_O_2_ and P_v-a_CO_2_ can better differentiate the 28-day mortality outcome. This somewhat validates our theoretical assumption, as it indicates that Group A performed the best, D performed the worst, and B performed better than C. No comparable investigations have been conducted by the authors. To our knowledge, this is the first study to report such findings.

The four groups showed no differences in baseline lactate and S_v_O_2_ levels, and neither was a risk factor for the 28-day mortality. However, Ospina-Tascón et al. [9] found that lactate at T6 was an independent predictor of 28-day mortality, which is contrary to our study and may be due to the different choice of T0 (their T0 at successful pulmonary artery catheterization; our T0 at ICU admission). Lactate metabolic kinetics are slow; clinical monitoring readings lag hemodynamic changes, and tissue hypoxia may not be the primary cause of its elevation [34]. Within 72 h, lactate levels declined, with Group A showing a significantly greater reduction than C and D, but no difference from B. A high C_v-a_CO_2_/C_a-v_O_2_ may indicate hypoxia-driven lactate elevation, while a normal ratio indicates other causes. Data from Groups A and B suggest that even with adequate oxygen delivery, impaired perfusion can limit intracellular oxygen use, raising lactate levels. In critically ill patients, factors like hyperglycemia, catecholamines, lactate-containing fluids, high metabolism, and delayed clearance can elevate lactate. S_v_O_2_ may be excessively high or low to indicate tissue hypoxia [35]. Our findings demonstrate that the correlation between lactate or S_v_O_2_ and the incidence of clinical outcomes is poor. These factors may have influenced the outcomes of this study.

We monitored microcirculatory indicators and found connections between C_v-a_CO_2_/C_a-v_O_2_, P_v-a_CO_2_, and sPPV at T6, suggesting that C_v-a_CO_2_/C_a-v_O_2_ and P_v-a_CO_2_ reflect microcirculatory adequacy, consistent with the findings of Ospina-Tascón et al. [32]. Our study indicated that C_v-a_CO_2_/C_a-v_O_2_ (higher *r* and R^2^ values) connected better with sPPV than P_v-a_CO_2_, despite both having low R^2^ values, possibly due to the microcirculatory system’s intricacy and many influencing factors. The microcirculatory disturbances and tissue perfusion abnormalities in septic shock may be coupled with cellular and mitochondrial dysfunction, and that this coupling merely reflects an adaptive change [36]. This may also explain why Group B outperformed C. In univariate Cox regression, among the microcirculatory indicators, only sPPV predicted 28-day mortality; However, it was not an independent predictor in multivariate analysis, suggesting that its impact on C_v-a_CO_2_/C_a-v_O_2_ and P_v-a_CO_2_ may indirectly affect mortality. sPPV, as the most promising and reproducible indicator of microcirculatory tissue perfusion in patients [37], has been less studied in relation to 28-day mortality. At T6, patients with sPPV < 0.82 and MFI < 2.4 (Group D) exhibited the highest mortality rate (46.7%). These thresholds may facilitate early identification of high-risk patients, even when macrocirculatory targets are achieved. This could prompt timely escalation of therapy, including individualized fluid management and vasodilator use, to address persistent microcirculatory dysfunction. Notably, recent multicenter randomized controlled research [37] found no shock patient survival benefit from incorporating microcirculatory perfusion markers into the treatment.

In this study, the calculation formula for C_v-a_CO_2_/C_a-v_O_2_ is complex; however, the associated data and P_v-a_CO_2_ are readily available in clinical settings. As these parameters are part of the routine monitoring of S_v_O_2_ and arterial blood gases, obtaining them does not impose extra trauma or costs on the patient. We acknowledge the limitations of this study, as it measured S_v_O_2_, P_v-a_CO_2_, and C_v-a_CO_2_/C_a-v_O_2_ using superior vena cava blood instead of mixed venous blood, which better represents systemic tissue VO_2_ and VCO_2_, potentially influencing the results. Moreover, current studies on the consistency of these two sampling sites have primarily focused on S_v_O_2_ rather than PCO_2_. Few patients in the study had CO measured by thermodilution because it is expensive and invasive. Bedside ultrasound monitoring of CO was not compared as an observational indicator because it is influenced by many factors. CO was not compared to an observational indicator. The lack of similar studies made the sample size estimation challenging, which could have prevented us from correctly answering our research hypotheses correctly. This observational pilot study required flexibility to explore novel findings; hence, the protocol was not pre-registered or previously published. Finally, the findings of this study require validation through multicenter, large-sample studies to assess the generalizability of C_v-a_CO_2_/C_a-v_O_2_ and P_v-a_CO_2_ as prognostic markers. Integrating the two parameters with established scoring systems or new biomarkers could enhance clinical decision-making for septic shock patients.

In recent years, the management of septic shock shifted towards dynamic assessment of macro- and micro-circulatory parameters and personalized resuscitation strategies. In septic shock, a state of macrocirculation-microcirculation uncoupling may exist. Therefore, while optimizing macrocirculatory parameters, it is crucial to integrate dynamic microcirculatory assessments for a comprehensive hemodynamic evaluation. Future research could focus on longitudinal monitoring of the microcirculation, investigating how changes in C_v-a_CO_2_/C_a-v_O_2_ and P_v-a_CO_2_ over time correlate with microcirculatory dynamics.

## Conclusions

The combined assessment of C_v-a_CO_2_/C_a-v_O_2_ and P_v-a_CO_2_ during the early stages of resuscitation demonstrates a significant association with mortality in septic shock patients. This combination could potentially serve as a resuscitation target and reflect microcirculatory perfusion in septic shock patients.

## Supplementary Information

Additional file 1.

## Data Availability

The datasets used and/or analyzed during the current study are available from the corresponding author on reasonable request.

## Abbreviations

C_v-a_CO_2_/C_a-v_O_2_: The ratio of central venous-to-arterial carbon dioxide content difference to arterial-to-venous oxygen content difference
P_v-a_CO_2_: The central venous-to-arterial carbon dioxide tension difference
ICU: Intensive care unit
T6: 6 Hours post-ICU admission
PPV: The proportion of perfused vessels for all
sPPV: The proportion of perfused vessels for *d* < 20 μm
MFI: Microvascular flow index
HI: Heterogeneity index
HR: Hazard ratio
95% CI: 95% Confidence interval
S_v_O_2_: Venous O_2_ saturation
DO_2_: Oxygen delivery
CO: Cardiac output
VCO_2_: CO_2_ production
VO_2_: O_2_ consumption
RQ: Respiratory quotient
P_v-a_CO_2_/C_a-v_O_2_: The ratio of central venous-to-arterial carbon dioxide tension difference to arterial-to-venous oxygen content difference
SDF: Sidestream dark field
SOFA: Sequential organ failure assessment
MAP: Mean arterial pressure
CVP: Central venous pressure
STROBE: Strengthening the Reporting of Observational Studies in Epidemiology
CI: Cardiac index
S_cv_O_2_: Central venous O_2_ saturation
IBW: Ideal body weight
Pplat: Plateau pressure
PEEP: Positive end-expiratory pressure
FiO_2_: Fraction of inspiration O_2_
ARDS: Acute respiratory distress syndrome
P_a_O_2_: Partial pressure of O_2_ in arterial blood
P_a_CO_2_: Partial pressure of CO_2_ in arterial blood
CPOT: Critical-Care Pain Observation Tool
Hb: Hemoglobin
T0: Time 0
HR: Heart rate
VIS: Vasoactive-inotropic score
APACHE II: Acute Physiology and Chronic Health Evaluation II
TVD: Total vessel density
sVD: Vessel (*d* < 20 μm) density
PVD: Erfused vessel density
sPVD: Perfused vessel (*d* < 20 μm) density
L_p_: Total length of perfused vessels
L_v_: Lengths of all vessels
A_FOV_: Total analyzed field of view area
C_a_O_2_: Arterial oxygen content
C_v_O_2_: Venous oxygen content
C_v_CO_2_: Venous blood CO_2_ content
C_a_CO_2_: Arterial blood CO_2_ content
S_a_O_2_: Arterial oxygen saturation
S_v_O_2_: Venous oxygen saturation
P_v_O_2_: Venous oxygen partial pressure
pH: Potential of hydrogen
IQR: Interquartile range
ANOVA: Analysis of variance
BMI: Body mass index
CKD: Chronic Kidney Disease
CRRT: Continuous renal replacement treatment
AUC: Area under the curve
