# Supplementary material for: Combination of Cv-aCO2/Ca-vO2 and Pv-aCO2 as markers of resuscitation or microcirculation in patients with septic shock: a pilot study
Source: J Intensive Care. 2025 Jun 17;13:35. doi: 10.1186/s40560-025-00801-2 (PMC12172353; doi:10.1186/s40560-025-00801-2)
Supplement: Supplementary file 1 — Additional file 1. [file 40560_2025_801_MOESM1_ESM.docx]

**Combination of C_v-a_CO_2_/C_a-v_O_2_ and P_v-a_CO_2_ as markers of resuscitation** **or microcirculation in patients with septic shock: a pilot study**

Luping Cheng MD^1†^, Wenxin Wang MD^1†^, Xia Hu MD^1^, Chuanliang Pan MD^2*^

^1^North Sichuan Medical College, Nanchong, Sichuan, China.

^2^Department of Intensive Care Unit, The Third people’s Hospital of Chengdu, Affiliated Hospital of Southwest Jiaotong University, Chengdu, China.

^†^These authors contributed equally to this work.

^*^**Correspondence author:** Chuanliang Pan, **Email:** [cl.pan@foxmail.com](mailto:cl.pan@foxmail.com)

**Supplementary material**

Table S1…………………………………………………………………………….………2

Table S2………………………………………………………...……………………….….3

Table S3……..…………………………………………………………...…………….…...4

Fig. S1…………………………………………………………….…………………..…….5

Fig. S2……………………………………………………………….………………..…....6

Table S1 Alterations in lactate levels, SOFA and APACHE Ⅱ scores among the four groups within 72 hours.

| Variables | All patients  (n=105) | Group A  (n=24) | Group B  (n=21) | Group C  (n=30) | Group D  (n=30) | *P* |
| --- | --- | --- | --- | --- | --- | --- |
| lactate (mmol/L) |  | †  * | * |  |  |  |
| T6 | 5.2±2.0 | 5.1±1.7 | 4.8±1.8 | 5.2±2.3 | 5.5±2.2 | <0.001 |
| T24 | 4.8±1.7 | 4.0±1.6 | 4.2±1.4 | 5.2±1.2 | 5.5±1.9 |  |
| T48 | 3.8±1.4 | 3.1±1.4 | 3.3±1.3 | 3.9±1.2 | 4.5±1.5 |  |
| T72 | 3.3±1.4 | 2.9±1.2 | 3.0±1.3 | 3.5±1.3 | 3.7±1.6 |  |
| SOFA scores |  | †  * | * |  |  |  |
| T6 | 11.4±2.5 | 10.6±2.4 | 11.0±2.0 | 11.9±2.2 | 11.7±3.1 | <0.001 |
| T24 | 8.1±3.2 | 7.8±2.8 | 6.9±2.8 | 7.7±3.3 | 9.6±3.2 |  |
| T48 | 8.0±3.1 | 6.1±2.8 | 7.4±3.2 | 8.6±2.8 | 9.2±3.0 |  |
| T72 | 6.7±3.0 | 5.2±2.3 | 6.6±3.2 | 7.0±2.9 | 7.8±3.1 |  |
| APACHE Ⅱ scores |  | * | * |  |  |  |
| T24 | 22.9±4.9 | 22.2±5.9 | 23.8±4.0 | 22.7±4.6 | 23.0±5.0 | <0.001 |
| T48 | 20.6±5.3 | 18.4±5.2 | 18.8±3.2 | 21.5±5.1 | 22.7±6.0 |  |
| T72 | 18.9±4.6 | 16.8±3.7 | 17.7±3.9 | 18.4±3.7 | 21.8±5.3 |  |

†*P* < 0.05 vs. Group C; **P* < 0.05 vs. Group D.

Patients were separated into four groups according to C_v-a_CO_2_/C_a-v_O_2_ and P_v-a_CO_2_ measured at T6: Group A, C_v-a_CO_2_/C_a-v_O_2_ ≤ 1 and P_v-a_CO_2_ < 6 mmHg; Group B, C_v-a_CO_2_/C_a-v_O_2_ ≤ 1 and P_v-a_CO_2_ ≥ 6 mmHg; Group C, C_v-a_CO_2_/C_a-v_O_2_ > 1 and P_v-a_CO_2_ < 6 mmHg; Group D, C_v-a_CO_2_/C_a-v_O_2_ > 1 and P_v-a_CO_2_ ≥ 6 mmHg.

T6, at 6 hours post-ICU admission; T24, at 24 hours post-ICU admission; T48, at 48 hours post-ICU admission; T72, at 72 hours post-ICU admission. SOFA, sequential organ failure assessment; APACHE Ⅱ, acute physiology and chronic health evaluation Ⅱ.

**Table S2** Univariate and multivariate Cox regression for predictors of 28-day mortality.

| **Variables** | **Univariate** | | | **Multivariate** | | |
| --- | --- | --- | --- | --- | --- | --- |
|  | HR | 95%CI | *P* | HR | 95%CI | *P* |
| Age | 1.04 | 1.01-1.08 | **0.004** | 1.05 | 1.02-1.09 | **0.001** |
| Male | 0.94 | 0.45-1.95 | 0.940 | - | - | - |
| BMI | 1.01 | 0.94-1.09 | 0.788 | - | - | - |
| Source of infection | 0.99 | 0.78-1.25 | 0.923 | - | - | - |
| Medical /surgical | 0.82 | 0.34-2.02 | 0.669 | - | - | - |
| Diabetes | 0.73 | 0.35-1.52 | 0.400 | - | - | - |
| Hypertension | 1.51 | 0.69-3.32 | 0.303 | - | - | - |
| CKD | 0.67 | 0.20-2.21 | 0.511 | - | - | - |
| Cardiopathy | 0.94 | 0.33-2.71 | 0.911 | - | - | - |
| T6, CRRT | 1.15 | 0.44-3.02 | 0.774 | - | - | - |
| Fluids before T6 | 1.00 | 0.99-1.00 | 0.617 | - | - | - |
| T6, BT | 1.12 | 0.79-1.59 | 0.519 | - | - | - |
| T6, MAP | 1.06 | 0.95-1.17 | 0.283 | - | - | - |
| T6, CVP | 0.99 | 0.82-1.20 | 0.921 | - | - | - |
| T6, Heart rate | 0.98 | 0.96-1.01 | 0.165 | - | - | - |
| T6, PEEP | 0.94 | 0.80-1.10 | 0.427 | - | - | - |
| T6, FiO_2_ | 1.21 | 0.21-6.88 | 0.827 | - | - | - |
| T6, Hb | 0.98 | 0.96-1.01 | 0.114 | - | - | - |
| T6, VIS | 0.99 | 0.94-1.04 | 0.636 | - | - | - |
| T6, S_v_O_2_ | 5.13 | 0.33-79.12 | 0.242 | - | - | - |
| T6, lactate | 0.95 | 0.79-1.15 | 0.614 | - | - | - |
| T6, SOFA | 1.06 | 0.91-1.23 | 0.450 | - | - | - |
| T6, C_v-a_CO_2_/C_a-v_O_2_ | 1.80 | 1.17-2.77 | **0.008** | 1.67 | 1.03-2.69 | **0.036** |
| T6, P_v-a_CO_2_ | 1.16 | 1.03-1.30 | **0.014** | 1.13 | 1.00-1.27 | **0.043** |
| T24, APACHE Ⅱ | 1.01 | 0.94-1.09 | 0.733 | - | - | - |
| PPV | <0.01 | 0-1.20 | 0.058 | - | - | **-** |
| sPPV | <0.01 | 0-0.21 | **0.010** | 0.06 | 0-14.51 | 0.311 |
| MFI | 0.37 | 0.10-1.46 | 0.374 | - | - | **-** |
| HI | 2.06 | 0.76-5.63 | 0.157 | - | - | **-** |
| sVD | 0.95 | 0.86-1.06 | 0.375 | - | - | **-** |
| TVD | 0.95 | 0.89-1.02 | 0.153 | - | - | **-** |
| PVD | 0.94 | 0.87-1.01 | 0.070 | - | - | **-** |
| sPVD | 0.92 | 0.82-1.03 | 0.153 | - | - | **-** |

Candidate predictors with statistically significant differences (*p* < 0.05) in univariate Cox regression were included in multivariate Cox regression. Hazard ratio (HR) and 95% Confidence Interval (95% CI) are reported. Statistically significant *P* values (< 0.05) are highlighted in bold.

**Table S3** Microcirculatory variables for the four groups at T6.

| Variables | All patients  (n=105) | Group A  (n=24) | Group B  (n=21) | Group C  (n=30) | Group D  (n=30) | *P* |
| --- | --- | --- | --- | --- | --- | --- |
| PPV | 0.94 (0.89, 0.98) | 0.98 (0.97, 0.99) ‡†* | 0.95 (0.92, 0.97) * | 0.92 (0.89, 0.96) | 0.88 (0.84, 0.94) | <0.001 |
| sPPV | 0.90 (0.86, 0.94) | 0.94 (0.90, 0.98) †* | 0.92 (0.90, 0.95) †* | 0.89 (0.86, 0.91) | 0.82 (0.78, 0.90) | <0.001 |
| MFI | 2.6 (2.4, 2.8) | 2.9 (2.7, 3.0)†* | 2.7 (2.5, 2.8) * | 2.6 (2.6, 2.7)* | 2.4 (2.2, 2.4) | <0.001 |
| HI | 0.74 (0.36, 0.83) | 0.34 (0, 0.42) †* | 0.37 (0.36, 0.77) * | 0.74 (0.38, 0.77) * | 0.85 (0.74, 0.92) | <0.001 |
| TVD (mm/mm^2^) | 23.8 (20.3, 29.2) | 23.9 (20.8, 31.1) | 23.3 (19.5, 27.4) | 24.3 (19.6, 29.6) | 23.8 (20.9, 28.8) | 0.669 |
| sVD (mm/mm^2^) | 13.0 (11.0, 16.0) | 13.0 (10.2, 15.0) | 12.0 (10.0, 15.0) | 13.5 (10.8, 17.0) | 13.0 (11.8, 16.0) | 0.565 |
| PVD (mm/mm^2^) | 22.1 (18.6, 27.5) | 23.6 (20.2, 30.8) | 21.8 (18.1, 26.4) | 22.7 (18.1, 26.9) | 21.6 (17.3, 26.5) | 0.208 |
| sPVD (mm/mm^2^) | 12.0±3.5 | 12.5±3.7 | 11.7±3.4 | 12.5±4.0 | 11.5±3.0 | 0.643 |

‡*P* < 0.05 vs. Group B; †*P* < 0.05 vs. Group C; **P* < 0.05 vs. Group D.

Patients were separated into four groups according to C_v-a_CO_2_/C_a-v_O_2_ and P_v-a_CO_2_ measured at T6: Group A, C_v-a_CO_2_/C_a-v_O_2_ ≤ 1 and P_v-a_CO_2_ < 6 mmHg; Group B, C_v-a_CO_2_/C_a-v_O_2_ ≤ 1 and P_v-a_CO_2_ ≥ 6 mmHg; Group C, C_v-a_CO_2_/C_a-v_O_2_ > 1 and P_v-a_CO_2_ < 6 mmHg; Group D, C_v-a_CO_2_/C_a-v_O_2_ > 1 and P_v-a_CO_2_ ≥ 6 mmHg.


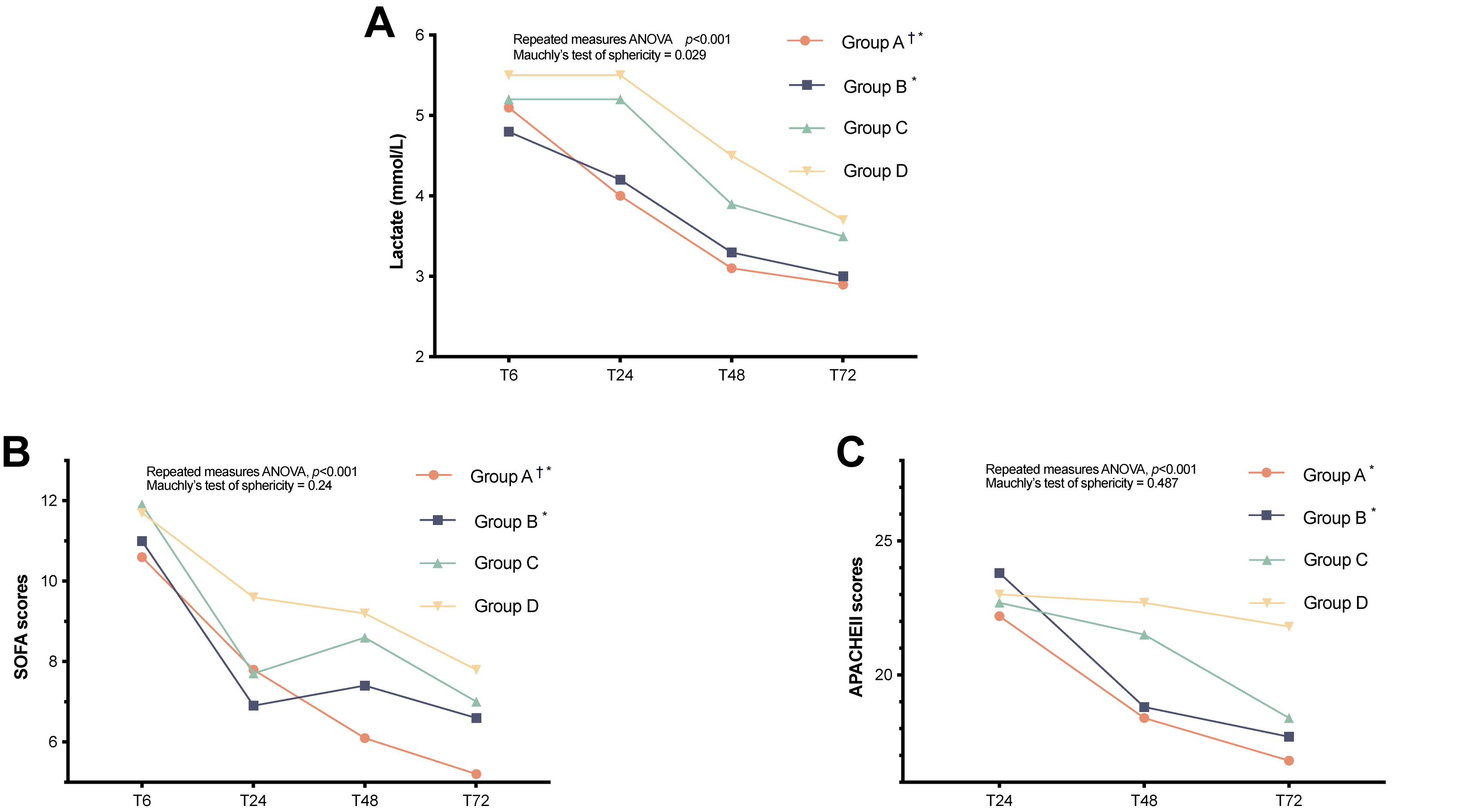


**Fig. S1** Trends in lactate levels, SOFA scores, and APACHE Ⅱ scores among the four groups within 72 hours. †*P* < 0.05 vs. Group C; **P* < 0.05 vs. Group D.

Patients were separated into four groups according to C_v-a_CO_2_/C_a-v_O_2_ and P_v-a_CO_2_ measured at T6: Group A, C_v-a_CO_2_/C_a-v_O_2_ ≤ 1 and P_v-a_CO_2_ < 6 mmHg; Group B, C_v-a_CO_2_/C_a-v_O_2_ ≤ 1 and P_v-a_CO_2_ ≥ 6 mmHg; Group C, C_v-a_CO_2_/C_a-v_O_2_ > 1 and P_v-a_CO_2_ < 6 mmHg; Group D, C_v-a_CO_2_/C_a-v_O_2_ > 1 and P_v-a_CO_2_ ≥ 6 mmHg. T6, at 6 hours post-ICU admission; T24, at 24 hours post-ICU admission; T48, at 48 hours post-ICU admission; T72, at 72 hours post-ICU admission; SOFA, sequential organ failure assessment; APACHE Ⅱ, acute physiology and chronic health evaluation Ⅱ.

**
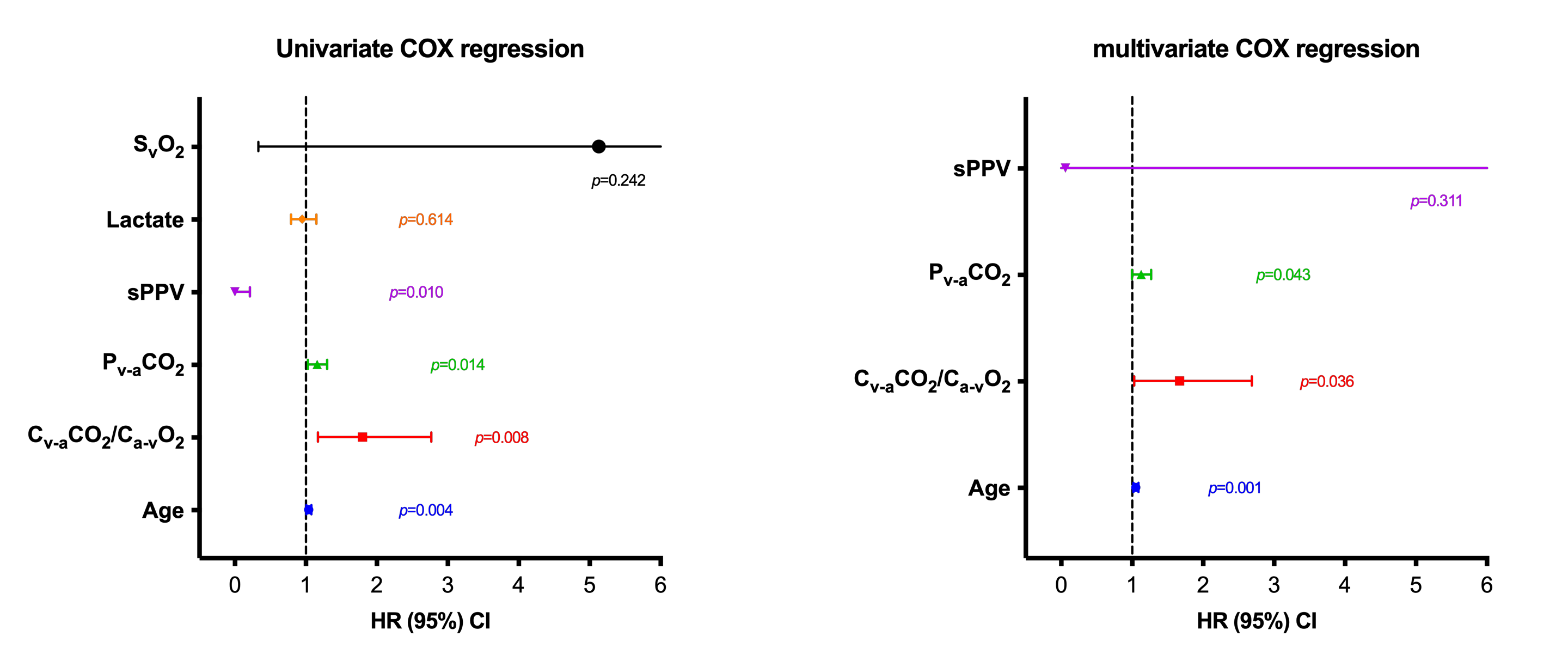
**

**Fig. S2** Forest map visualizing Cox univariate and multivariate regression for predictors of 28-day mortality. The horizontal lines indicating the 95% confidence intervals. The dashed vertical line represents the null effect. Each point (or square, circle, triangle, etc.) on the line segment represents the effect estimate.
